# Retraction: Long noncoding RNA HOXA-AS2 promotes non-small cell lung cancer progression by regulating miR-520a-3p

**DOI:** 10.1042/BSR-2019-0283_RET

**Published:** 2022-10-07

**Keywords:** HOXA-AS2, Long non-coding RNA, miR-520a-3p, non-small cell lung cancer, proliferation

This Retraction follows an Expression of Concern relating to this article previously published by Portland Press. This article is being retracted from Bioscience Reports at the request of the authors following receipt of a notification from a reader, alerting the Editorial Office to duplications that occur across multiple papers with no common authors. Similarities exist between Figure panel 3b and Figure panel 3b of the retracted article ‘Long noncoding RNA 00460 (LINC00460) promotes glioma progression by negatively regulating miR-320a’ (DOI: 10.1002/jcb.28232), as well as a vertically flipped version of Figure panel 3d of the article ‘STAT3-mediated upregulation of lncRNA HOXD-AS1 as a ceRNA facilitates liver cancer metastasis by regulating SOX4’ (DOI: 10.1186/s12943-017-0680-1). The authors wish to retract the article. The Editor-in-Chief and Editorial Board agree with the Retraction.

